# Bacteriophage-encoded shiga toxin gene in atypical bacterial host

**DOI:** 10.1186/1757-4749-3-10

**Published:** 2011-07-07

**Authors:** Veronica Casas, Gerico Sobrepeña, Beltran Rodriguez-Mueller, Justine AhTye, Stanley R Maloy

**Affiliations:** 1 San Diego State University 5500 Campanile Drive San Diego, California 92182-4614 USA; 2 Center for Microbial Sciences 5500 Campanile Drive San Diego, California 92182-4614 USA

## Abstract

**Background:**

Contamination from fecal bacteria in recreational waters is a major health concern since bacteria capable of causing human disease can be found in animal feces. The Dog Beach area of Ocean Beach in San Diego, California is a beach prone to closures due to high levels of fecal indicator bacteria (FIB). A potential source of these FIB could be the canine feces left behind by owners who do not clean up after their pets. We tested this hypothesis by screening the DNA isolated from canine feces for the bacteriophage-encoded *stx *gene normally found in the virulent strains of the fecal bacterium *Escherichia coli*.

**Results:**

Twenty canine fecal samples were collected, processed for total and bacterial fraction DNA, and screened by PCR for the *stx *gene. The *stx *gene was detected in the total and bacterial fraction DNA of one fecal sample. Bacterial isolates were then cultivated from the *stx*-positive fecal sample. Eighty nine of these canine fecal bacterial isolates were screened by PCR for the *stx *gene. The *stx *gene was detected in five of these isolates. Sequencing and phylogenetic analyses of 16S rRNA gene PCR products from the canine fecal bacterial isolates indicated that they were *Enterococcus *and not *E. coli*.

**Conclusions:**

The bacteriophage-encoded *stx *gene was found in multiple species of bacteria cultivated from canine fecal samples gathered at the shoreline of the Dog Beach area of Ocean Beach in San Diego, California. The canine fecal bacteria carrying the *stx *gene were not the typical *E. coli *host and were instead identified through phylogenetic analyses as *Enterococcus*. This suggests a large degree of horizontal gene transfer of exotoxin genes in recreational waters.

## Background

Each year millions of people flock to the beaches of San Diego County for fun in the sun, sand, and surf. Monitoring water quality at these beaches is therefore extremely important to limit the possibility of illness from contact with these recreational waterways. Through San Diego County's Department of Health (DEH), the Ocean and Bay Recreational Water Program (OBRWP) is responsible for monitoring the water quality of San Diego's recreational waterways [1]. Most beaches in San Diego County are relatively clean, with little to no advisories or beach closures, but there are also areas prone to advisories and/or beach closures because of known sources of pollution [1,2]. Posted warning signs, a daily water quality report hotline, and an internet podcast are some of the ways the OBRWP informs the public about general advisories or beach closures they have issued as a result of elevated bacterial levels in the water [1]. Typical sources of bacterial contamination at San Diego County beaches include urban runoff from storm drains and rivers, animal waste, human activities, and sewage.

Contamination from human and animal feces is of particular concern because infectious disease agents like bacteria, viruses, and protozoa are shed in the feces of infected individuals [3]. *Vibrio cholera *(cholera), *Salmonella *spp. (typhoid fever, gastroenteritis), and *Shigella *spp. (shigellosis) are bacteria found in feces and cause gastrointestinal disease [3]. Some pathogenic viruses that can be transmitted through use of recreational waterways include enteroviruses, hepatitis A viruses (HAV), polioviruses, coxsackie viruses, echoviruses, rotaviruses, and Norwalk viruses [4-9]. They cause a broad range of disease, not necessarily gut related, from hepatitis to polio to acute viral gastroenteritis [5,9]. *Cryptosporidium, Giardia*, and *Entamoeba *are common protozoan pathogens that cause cryptosporidiosis, giardiasis, and amoebic dysentery, respectively [3,4,9-13].

Though these organisms are common sources of waterborne diseases, their presence in these waters is not easily determined. As a result, presence of fecal indicator organisms has been accepted as the factor in determining water quality. Ideally, fecal indicator organisms would be good predictors of fecal contamination, and therefore good predictors of the potential for human illness. These fecal indicator organisms would be present whenever the pathogens were present, would survive in the environment as long as the pathogen was present, and would be easily detectable and cultivable from environmental samples [3]. Unfortunately, the relationship between presence of fecal indicator organisms, bacterial and viral pathogens, and actual fecal contamination is poorly understood. This leaves public health agencies at a disadvantage in determining the potential risk to the public's health when using recreational waterways.

The various San Diego County water quality monitoring laboratories currently use culturing assays (membrane filtration, multiple-tube fermentation, Colilert 18^®^, Enterolert^®^) to assess presence of fecal indicator organisms--namely coliforms [1,14]. These assays take advantage of metabolic and enzymatic properties common to coliform bacteria [3]. Many studies have been performed to evaluate if fecal indicator organisms serve as a good proxy for monitoring water quality. Comparing cultivation tests for coliforms to molecular tests for other pathogenic bacteria and viruses, the studies showed mixed results--mostly indicating that relationships depend upon the water type, exposed population, and weather conditions (see [15]for a review) [7,15-19].

It is important to be able to have a broader view of the bacterial and viral community present in recreational waters because of their potential to cause human disease. This environment may provide selection for new virulence traits that could be missed by current methods. One mechanism for acquiring new virulence traits is through horizontal gene transfer (HGT). Bacteriophage are common mediators of this genetic exchange and they often carry genes that code for virulence factors. Since the discovery that phage β from *Corynebacteria diphtheria *carried the gene for diphtheria toxin and was responsible for the virulence traits of *C. diphtheria*, many more bacteriophage-encoded virulence genes have been discovered [20,21]. Some of these include the shiga toxin (*stx*) gene of *Escherichia coli *O157:H7 species, the cholera toxin gene (*ctx*) carried by ctxϕ of *Vibrio cholera*, and the *Staphylococcus *enterotoxin A (*sea*) gene of *Staphylococcus aureus *[22-24]. Considering the high concentrations of bacteriophage and bacteria in the ocean, transduction frequencies in the World's oceans has been estimated to be as high as 20 million billion transduction events per second [25]. It is therefore important to understand the interactions between bacteria and bacteriophage in recreational waters and the potential for evolution of novel pathogens within these environments, to be able to better understand the risk to the public's health when using these waters.

## Materials and Methods

### Canine feces sampling strategy

**Figure 1:**
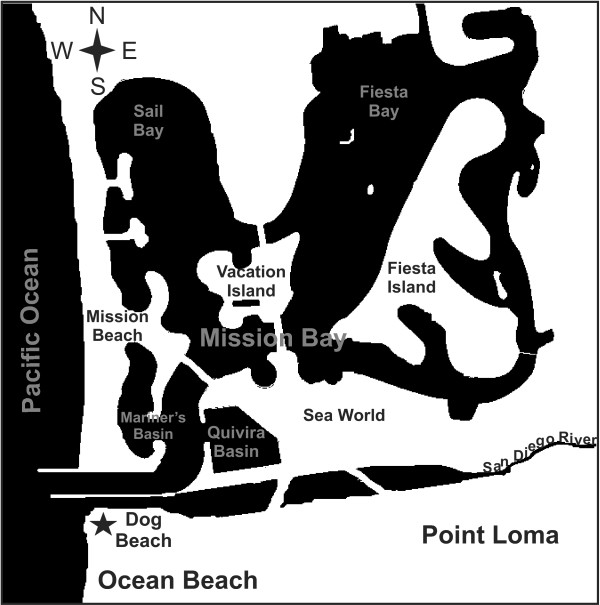
**Map of sampling location and surrounding waterways**. The Dog Beach area of Ocean Beach in San Diego, CA and surrounding waterways. Dog Beach is one of a few beaches in San Diego where people are allowed to bring their canine companions. The San Diego River and popular Mission Bay recreational waterways empty into the Pacific Ocean at this location. The black star indicates the Dog Beach area where fecal samples were gathered.

Twenty canine fecal samples were gathered at the water's edge at the Ocean Beach Dog Beach in San Diego, California (Figure 1). The fecal samples were identified visually in the sand and gathered carefully with gloved hands using new disposable sandwich bags. The samples varied in size, color, consistency, and dampness. Samples were gathered and immediately returned to the lab for processing.

### Extraction of DNA from canine fecal samples

Total DNA was extracted from 1.0 g canine fecal samples using the MoBio UltraClean Soil DNA kit (Carlsbad, CA) maximum yield protocol. A previously described method was modified and used to extract bacterial fraction DNA from the canine fecal samples [26,27]. Briefly, fecal samples were suspended in 1:1 (w/v) 1X storage media (SM) buffer in a 50 ml conical vial and mixed overnight on an orbital shaker set at 100 rpm [27]. The samples were then centrifuged at 11,000 × *g *to pellet the biosolids. The supernatant was then filtered through a 0.2 μm Sterivex^® ^filter to capture the bacteria. The filter was then aseptically removed from its housing and DNA was extracted from the bacteria trapped on the filter using the MoBio UltraClean Soil DNA kit (Carlsbad, CA) maximum yield protocol.

### Exotoxin and 16S rRNA gene PCRs and sequencing of PCR products

Exotoxin-specific PCR was performed on the total and bacterial fraction DNA of the canine fecal samples. The primers and PCR conditions used in this study are as described previously [26]. Exotoxin-specific and 16S rRNA gene colony PCRs were performed on 89 canine fecal bacterial isolates, as described previously [26]. Five microliters of the bacterial isolate suspension cultivated from the canine feces was used as template in the PCRs. To control against contamination by PCR products, amplification and all subsequent downstream procedures were carried out in an entirely separate laboratory in a separate building from where DNA extraction and PCR assay set up procedures were performed. Separate and dedicated equipment and reagents were maintained in each laboratory. Also a negative control was included in each PCR assay performed.

The *stx *and 16S rRNA gene PCR products from the canine fecal bacterial isolates were gel purified using the MoBio UltraClean GelSpin kit (Carlsbad, CA) to prepare for sequencing. Sequencing of the *stx *and 16S rRNA gene PCR products was performed by the SDSU MicroChemical Core facility using the ABI Prism^® ^3100 capillary electrophoresis DNA sequencer.

### Cultivation of bacterial isolates from canine fecal samples

Canine fecal samples were re-suspended 1:1 (w/v) in 1X SM buffer in a 50 ml conical vial. The samples were mixed overnight on an orbital shaker set at 100 rpm. The samples were then centrifuged at 11,000 × *g *to pellet the biosolids. One hundred microliters of serial 10-fold dilutions of the supernatant were plated onto Luria Bertani (LB) agar plates to allow for isolation of single colonies. All plates were incubated at room temperature for 2-3 days until colonies were visible. Isolates were then sub-cultured into 96-well plates containing 150 μl LB broth with 15% glycerol. These sub-cultured isolates were then grown for another 2-3 days with aeration. Isolates were stored in the 96-well plates at 4°C until tested in the exotoxin-specific and 16S rRNA gene PCRs. Isolates were then placed at -80°C for permanent storage.

### Bioinformatic analyses of *stx *and 16S rRNA gene sequences

The *stx *PCR product sequences were identified by BLASTN alignment against the GenBank non-redundant nucleotide database [28,29]. The 16S rRNA gene sequences were de-replicated using FastGroup II [30]. The representative 16S rRNA gene FastGroup II sequences were used for the subsequent phylogenetic analyses. The 16S rRNA gene sequences were identified taxonomically by using the Ribosomal Database Project (RDP) Classifier [31] and phylogenetic trees were also generated as a means of classifying the canine fecal bacterial isolates. The *stx *sequences were also grouped according to the FastGroup II analyses and analyzed phylogenetically to visualize their relationship to other known *stx *sequences. The DNAPARS DNA parsimony program was used to generate the phylogenetic trees of both the 16S rRNA gene and *stx *PCR product sequences [32]. The bootstrap method with 1000 replicates, 989 steps, at 580 sites was performed for the 16S rRNA gene sequence analyses. For the *stx *gene sequence analyses, the bootstrap method with 1000 replicates, 2822 steps, at 1278 sites was performed. Consensus trees were used to represent the data and those groups at a relative frequency less than 10% were not shown.

## Results

### Shiga toxin (*stx*) gene detected in total and bacterial fraction DNA and bacterial isolate cultivated from canine feces

**Figure 2:**
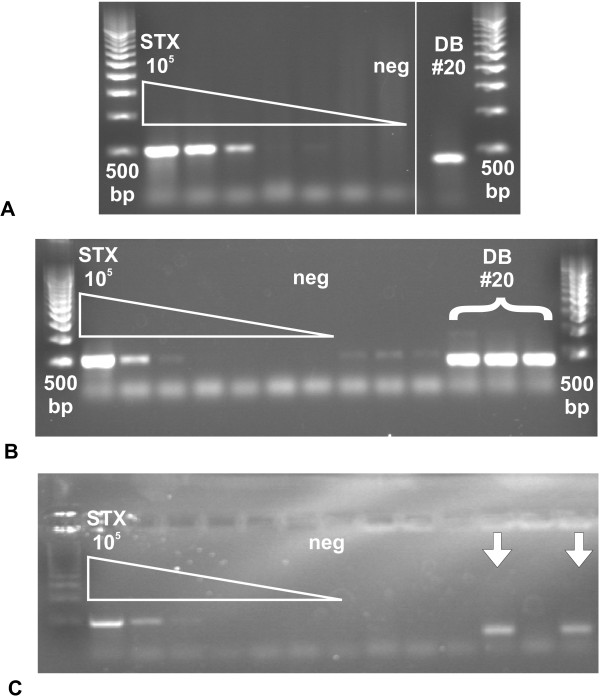
**Agarose gel picture of *stx*-specific PCR on canine fecal DNA and canine fecal isolates**. A) Initial *stx*-specific PCR screening of DNA extracted from canine fecal samples, B) *stx*-specific PCR on bacterial DNA extracted from canine fecal samples (n = 3, 10 μl and 20 μl loaded), and C) *stx*-specific PCR showing two of the five positive results from the cultured bacterial isolates. White arrows indicate the *stx*-positive isolates. White triangle is *stx *standard curve from 10E5 copies ml^-1 ^to one copy ml^-1^, neg = negative control, DB #20 = Dog Beach fecal sample #20, 500 bp = 500 base pair DNA ladder. Negative controls consistently had no detectable PCR products.

Twenty canine fecal samples were gathered from the shoreline at the Ocean Beach Dog Beach in San Diego, California. Total and bacterial fraction DNA was extracted from the fecal samples and screened for the *stx *gene by *stx*-specific PCR. The *stx *gene was detected in both the total and bacterial fraction DNA (Figure 2A and 2B, respectively). Bacterial isolates were cultivated from the canine fecal sample where the *stx *gene was detected. Eighty nine bacterial isolates were screened for the *stx *gene and it was detected in five of these isolates (Figure 2C). No PCR products were detected in the negative controls.

### Phylogenetic analyses of *stx *PCR product

**Figure 3:**
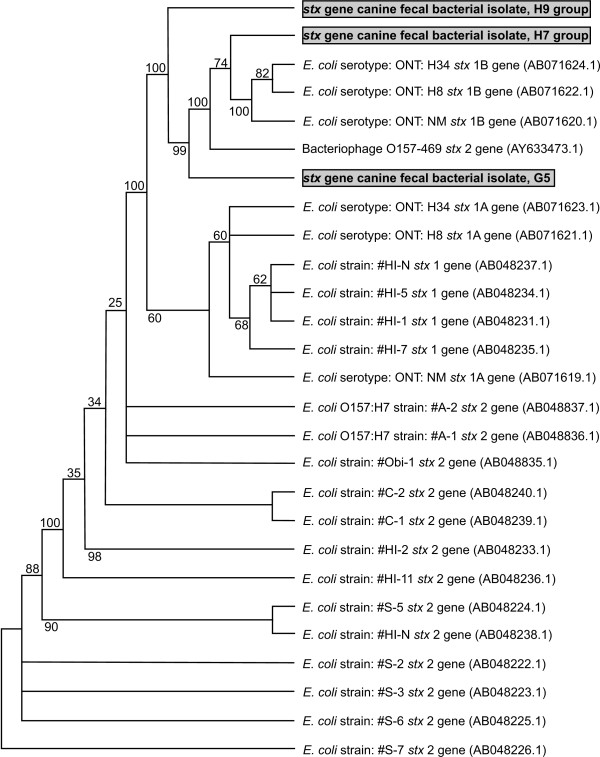
**Phylogenetic analysis of *stx *gene PCR product of total DNA**. The DNAPARS DNA parsimony program was used to generate the phylogenetic trees of the representative *stx *PCR product sequences [32]. The bootstrap method with 1000 replicates, 2822 steps, at 1278 sites was performed. The consensus tree is shown. The three canine fecal bacterial isolate groups are indicated by the gray boxes. GenBank accession numbers are indicated in parentheses.

The *stx *PCR products generated from the three FastGroup II representative canine fecal bacterial isolate groups were purified and sequenced (designated group 1-3, see 16S rRNA gene sequence results below for description). A BLASTN alignment of the sequences to the GenBank non-redundant nucleotide database was performed and they were confirmed to be the *stx *gene. A phylogenetic analysis of the *stx *sequences was also performed [28,29]. The three representative *stx *gene sequences were grouped with known *E. coli *O157:H7 *stx *gene sequences (Figure 3). The *stx *gene from groups 2 and 3 clustered together. The bootstrap values for these groupings were greater than 74.

### Canine fecal bacterial isolates carrying *stx *gene identified as Enterococcus

**Figure 4:**
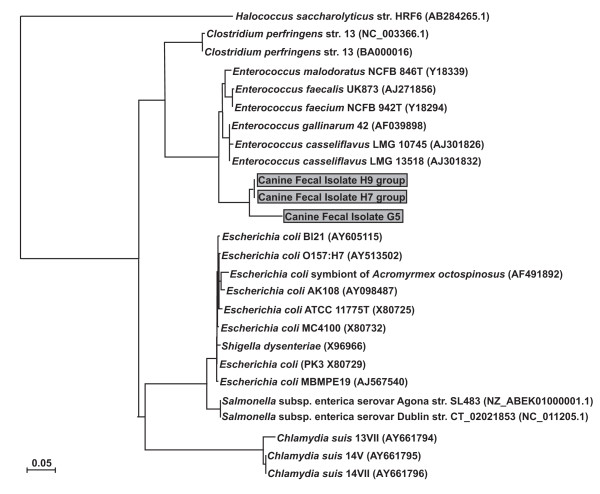
**Phylogenetic analysis of 16S rRNA gene PCR product from canine fecal bacterial isolates**. The DNAPARS DNA parsimony program was used to generate the phylogenetic trees of the three representative 16S rRNA gene PCR product sequences [32]. The bootstrap method with 1000 replicates, 989 steps, at 580 sites was performed. The consensus tree is shown. The three canine fecal bacterial isolate groups are indicated by the gray boxes. GenBank accession numbers are indicated in parentheses.

The *stx *gene was detected in five of the 89 bacterial isolates screened by *stx*-specific PCR (designated G516S.STX, H616S.STX, H716S.STX, H816S.STX, H916S.STX). A 16S rRNA gene PCR was performed on the five bacterial isolates. The resulting PCR product was purified and sequenced. The five 16S rRNA gene sequences were de-replicated using FastGroup II [30] and three distinct groups were identified (group 1 = G516S.STX; group 2 = H716S.STX, H816S.STX; group 3 = H616S.STX, H916S.STX). Representative sequences from these groups were utilized to molecularly identify the canine fecal bacterial isolates. First, the 16S rRNA gene sequences were identified taxonomically by using the Ribosomal Database Project (RDP) Classifier [31]. The RDP Classifier identified the three groups as belonging to the genus *Enterococcus*. To confirm this classification, a phylogenetic tree was generated (Figure 4). These phylogenetic analyses confirmed that the canine fecal isolates belonged to the genus *Enterococcus*.

## Conclusions

The beaches of San Diego, California are a popular destination for residents and tourists alike. Some of these beaches are designated as canine-friendly where people and their canine pets are allowed to enjoy the recreational waters together. One of these beaches is the Ocean Beach Dog Beach. This location is unique because not only is it a dog beach, but it is also an outlet into the Pacific Ocean for the San Diego River and the Mission Bay recreational waterways. As a result, this area is impacted by pollution and often closed for exceeding bacterial standards levels [1,2]. Important for water quality monitoring agencies is keeping the possibility of spread of infectious disease from bacteria and viruses relatively low. A paramount concern is pollution by human and animal waste because it carries disease-causing bacteria and viruses. With the close interaction of humans and animals at locations like Dog Beach, a potential reservoir for novel infectious disease pathogens may exist.

Exactly what kind of impact does the close interaction of humans and canines have on this environment and the microorganisms inhabiting it? Bacteria and their viruses (bacteriophage) are abundant in aquatic environments [33-37] and genetic exchange between these microorganisms occurs at a high frequency [25]. Some bacteriophage carry exotoxin genes that, when integrated into the bacterial chromosome, can transduce an avirulent bacterium to virulence. The influx of human- and animal-associated microorganisms to this environment may be providing a selective niche for the evolution of novel pathogens.

Many enteropathogenic microorganisms are found in animal feces and examples of the bacterial pathogens include *Salmonella *spp., *Shigella *spp., *Vibrio cholera*, and *Escherichia coli*. Certain strains of *Shigella *spp. and *E. coli *cause gastrointestinal illnesses and can carry the bacteriophage-encoded shiga toxin (*stx*) gene [38]. We hypothesized that bacteria cultivated from canine fecal samples collected from the Ocean Beach Dog Beach may contain the *stx *gene normally found in the fecal *Shigella *spp. or *E. coli*.

Canine fecal samples were collected from the shoreline of the Ocean Beach Dog Beach in San Diego, California and screened for the *stx *gene. The *stx *gene was detected in one of the 20 fecal samples. Bacterial isolates were cultivated from this fecal sample and the *stx *gene was detected in five of these isolates. Phylogenetic analyses of the 16S rRNA gene sequences from these isolates determined these isolates belonged to the *Enterococcus *genus and not the typical *E. coli *or *Shigella *spp. host. These results suggest a unique horizontal gene transfer event has occurred. One possibility for this event is that bacteriophage carrying the *stx *gene has a broad host range that allows it to infect *Enterococcus *spp. as well as *E. coli *and *Shigella *spp., or a genetic exchange has occurred between the *stx*-carrying-bacteriophage and a bacteriophage that infects *Enterococcus *spp.

Our results suggest that canine feces may be a reservoir for the bacteriophage-encoded *stx *gene and that this gene can be transferred to new bacterial hosts. This has implications in the development of new infectious diseases. A further, in depth assessment of the genomics of the bacterial and bacteriophage communities present in these recreational waterways and the potential fecal sources of contamination would shed more light on the extent to which horizontal gene transfer is occurring in these environments. Our study presents a first step in examining the reservoir of bacteriophage-encoded virulence genes present in animal waste and the potential impact exchange of these genes between atypical hosts may be having on the evolution of novel human pathogens.

## Competing interests

The authors declare that they have no competing interests.

## Authors' contributions

VC conceived of the study, designed the experiment and protocols, collected fecal samples, processed fecal samples, analyzed data, and drafted the manuscript. GS conceived of the study, collected fecal samples, processed fecal samples, executed PCR assays, performed culturing assays, and collected data. BRM performed the bioinformatic and phylogenetic analyses. JA processed fecal samples, managed cultured isolates, and executed PCR assays. SM provided advice in experimental design and reviewed and edited the manuscript. All authors read and approved the final manuscript.
